# False-Positive Elevation of Blood Gas Bilirubin in a Patient With Mixed Drug Overdose Involving Pregabalin and Diazepam Abuse

**DOI:** 10.7759/cureus.81387

**Published:** 2025-03-28

**Authors:** Patricia Cepeda, John Dixon, Ahmed Owies

**Affiliations:** 1 Critical Care and Anesthesiology, Epsom and St Helier University Hospitals NHS Trust, Carshalton, GBR; 2 Critical Care, Epsom and St Helier University Hospitals NHS Trust, Carshalton, GBR

**Keywords:** assay interference, bilirubin, case report, diazepam, overdose, pregabalin

## Abstract

Bilirubin measurements are commonly performed using a blood gas analyzer and are particularly useful in managing neonatal hyperbilirubinemia. This report presents a case of a white British man in his 40s who was treated for a mixed drug overdose involving cocaine, mirtazapine, fentanyl, codeine, methadone, morphine, and diazepam. His bilirubin levels measured on the blood gas analyzer were elevated at 63 µmol/L, whereas the normal range is 1.71-20.5 µmol/L. However, a formal laboratory bilirubin test returned a normal value of 21 µmol/L. Given the absence of jaundice or signs of liver disease, the unexpectedly high bilirubin reading on the blood gas analyzer prompted further investigation. Laboratory analysis involved scanning the absorbance of three serum samples received on the same day and comparing them with the absorbance of the blood gas analyzer. A significant peak at approximately 400 nm was detected in the first sample, corresponding to the wavelengths used for blood gas bilirubin measurement. Native bilirubin exhibits a broad absorbance spectrum between 350 and 500 nm, overlapping with the absorbance spectra of diazepam and pregabalin. This interference likely contributed to the false-positive bilirubin elevation. Currently, little is known about the relationship between false-positive bilirubin elevations and drugs of abuse. This case highlights potential causes of isolated bilirubin elevation, particularly interference from substances with overlapping absorbance spectra. While the interference is suspected, it has not been definitively proven. The findings underscore the limitations of arterial blood gas analysis and the potential for interference from other substances, particularly drugs of abuse. Clinicians should exercise caution when interpreting blood gas bilirubin concentrations in patients with a history of substance misuse. Consulting the laboratory is advisable when encountering unexpected discrepancies in bilirubin levels.

## Introduction

The association between the intake of sedative drugs, including diazepam and pregabalin, and false-positive hyperbilirubinemia is uncommon. According to Khandelwal et al., neither pregabalin nor diazepam therapy is linked to serum aminotransferase elevations. While clinically apparent liver injury has been reported for both drugs, such cases remain exceedingly rare [1].

Blood gas analyzers are increasingly used in critical care for rapid bilirubin assessment, aiding in timely clinical decision-making. As emphasized by Guerra Ruiz et al., while hyperbilirubinemia may indicate liver dysfunction, it does not always signify actual liver damage [2]. This is a crucial consideration for doctors managing patients in acute medical settings, including the ED and ICU. To ensure an accurate diagnosis, bilirubin level changes should be interpreted within the context of the patient’s history, the severity of elevation, and the pattern of associated biochemical abnormalities [2].

In this case, the initial blood gas sample showed a significantly elevated bilirubin concentration compared to the total serum bilirubin result from the laboratory. To determine the cause of this discrepancy, an investigation was conducted in the biochemistry laboratory by scanning the absorbance of three serum samples received on the same day and comparing them with the absorbance readings from the blood gas analyzers.

Given the substantial difference between the blood gas analyzer and laboratory serum bilirubin results, it is essential to maintain a high level of scrutiny and perform a thorough analysis to identify potential sources of error. This is critical to prevent inappropriate treatment for a patient with suspected hyperbilirubinemia but no jaundice or signs of liver disease. This study aims to explore the relationship between falsely elevated bilirubin levels in blood gas analysis and potential interference caused by drugs of abuse that the patient may have consumed.

## Case presentation

A man in his 40s was brought by ambulance to the ED after collapsing at home, where he was found by his partner. The estimated downtime was approximately 13 minutes. He had a three-day history of drowsiness and general malaise on a background of three weeks of worsening shortness of breath. On arrival, he was in type 2 respiratory failure with a Glasgow Coma Scale (GCS) score of 11. His family informed the medical team that he had packets of pregabalin and diazepam in his pocket. Due to his low GCS and inability to protect his airway, he was intubated.

**Figure 1:**
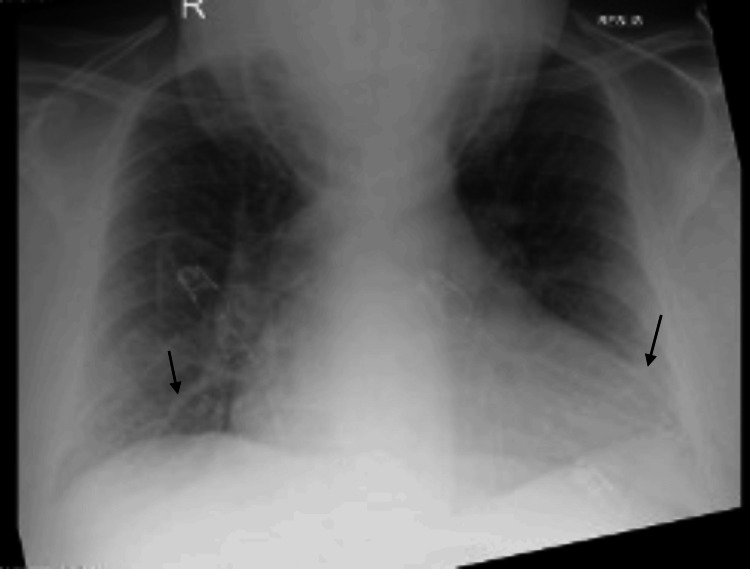
Initial chest X-ray demonstrating bibasal atelectasis (indicated by arrows)

**Figure 2:**
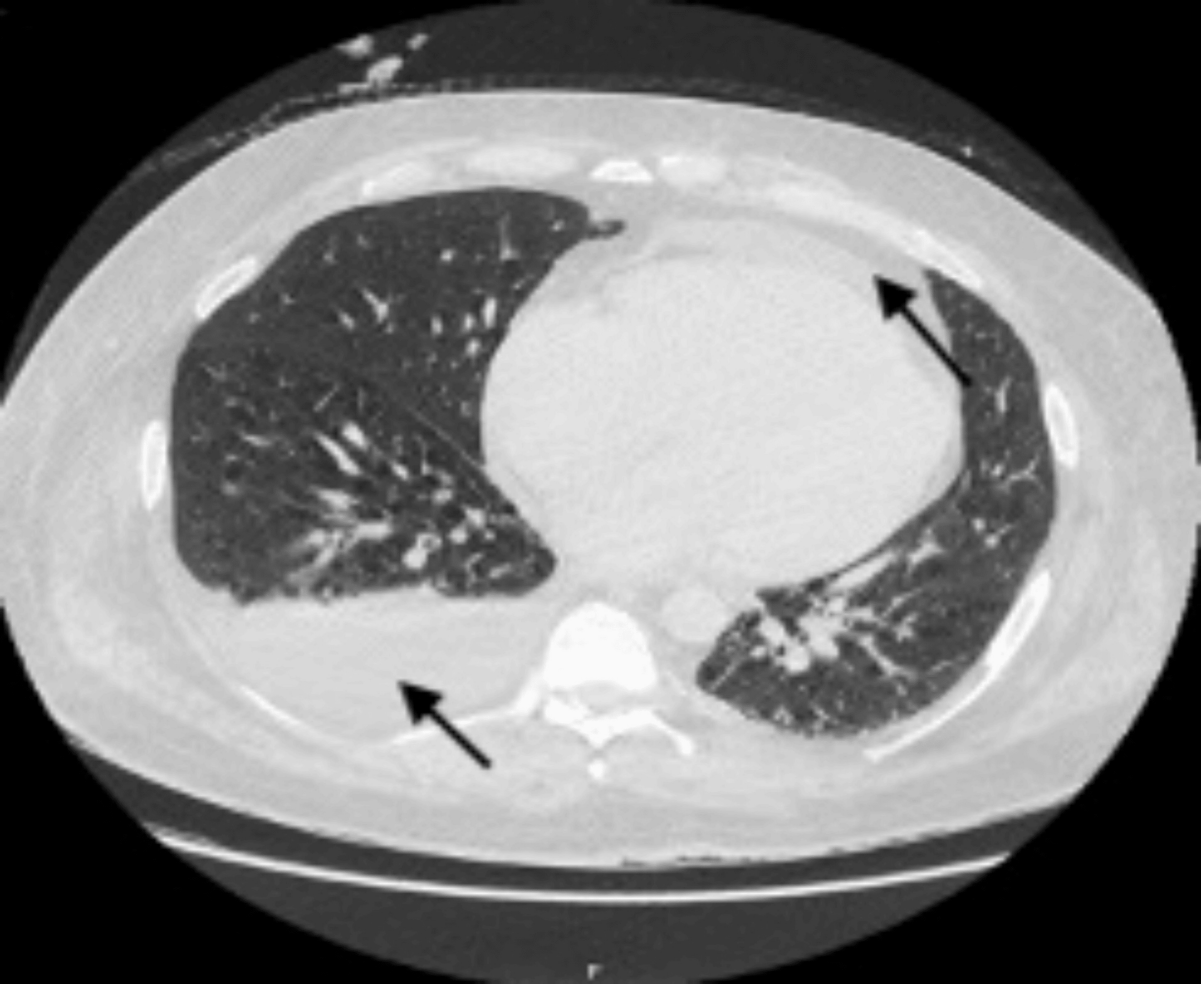
CT scan demonstrating right-sided pleural effusion and moderate pericardial effusion (indicated by arrows)

On examination, in addition to his reduced consciousness level, he had an estimated body mass of over 160 kg, corresponding to a BMI of approximately 50.5. There were no signs of jaundice, ascites, or gynecomastia. An initial chest X-ray revealed pulmonary congestion with upper lobe blood diversion and minor bibasal atelectasis (Figure 1). A CT scan of the abdomen and pelvis showed a moderate pericardial effusion, a right-sided pleural effusion (Figure 2), and moderate ascites. He was subsequently transferred to the ICU for respiratory support and further management.

The patient had a past medical history of depression, epilepsy, asthma, and post-traumatic stress disorder. He was a chronic smoker and reported abstaining from alcohol for more than 10 years following a stabbing incident, for which he underwent an exploratory laparotomy. There was no history of previous liver or biliary tract injury. His father noted a personality change after the incident, describing him as becoming more anxious and withdrawn. He had been prescribed mirtazapine, lamotrigine, and methadone for depression. His family history was unremarkable.

The patient’s inflammatory markers were elevated, and he required antibiotic treatment for presumed pneumonia with piperacillin-tazobactam, doxycycline, and clarithromycin. His inflammatory markers improved, and he completed a five-day course of antibiotics.

On admission, an arterial blood gas sample taken four hours after ingestion showed acute respiratory acidosis and an elevated bilirubin concentration of 63 µmol/L (Table 1). However, his laboratory bilirubin level was 21 µmol/L (normal: <25 µmol/L), with an ALT of 87 U/L (normal: <50 U/L). Other results were unremarkable. The rest of his initial workup is summarized in Table 2.

**Table 1:** Arterial blood gas results on admission

Variable	Result	Reference range
pH	7.19	7.35-7.45
pCO₂	13.3	4.70-6.40 kPa
pO₂	10.9	11.1-14.4 kPa
Total hemoglobin	194	135-175 g/L
SO₂	92	95-99%
F02Hb	88	94-98%
FCOHb	2.6	0.5-1.5%
FMetHb	1.4	0.0-1.5%
Sodium	139	136-146 mmol/L
Potassium	4.3	3.4-4.5 mmol/L
Chloride	95	98-106 mmol/L
Calcium	1.12	1.15-1.29 mmol/L
Lactate	0.6	0.5-1.6 mmol/L
Bilirubin	63	3-17 umol/L
HCO₃	28	21-28 mmol/L
FiO₂	80%	%
Patient’s temperature	37	°C

**Table 2:** Laboratory investigation results on admission

Variable	Result	Reference range
WBC (10⁹/L)	7.7	3.5-10.0
Hemoglobin (g/L)	187	130-170
Hematocrit (L/L)	0.635	0.40-0.50
Platelet (10⁹/L)	165	150-400
Sodium (mmol/L)	142	133-146
CRP (mg/L)	9.8	<5.0
Potassium (mmol/L)	4.7	3.5-5.3
Urea (mmol/L)	6.5	2.5-7.8
Creatinine (mmol/L)	80	20-107
Total bilirubin (µmol/L)	21	<25
ALP (U/L)	64	30-130
ALT (u/L)	87	1-50
Albumin (g/L)	30	35-50

A toxicology screen (Table 3) detected metabolites of cocaine, methadone, codeine, morphine, and diazepam in the patient’s system. Given the discrepancy between the bilirubin concentration reported by the blood gas analyzer (63 µmol/L) and the laboratory bilirubin measurement (21 µmol/L) and the absence of clinical signs of liver injury, we sought assistance from our colleagues in the biochemistry laboratory to investigate potential causes of this inconsistency. Additionally, we aimed to determine whether the elevated bilirubin detected in the blood gas analysis could be a false-positive result influenced by the sedative drugs the patient had taken.

**Table 3:** Urine toxicology screen results

Urine test for medicolegal drugs of abuse	Result
Pregabalin	Positive
Gabapentin	Negative
Promethazine	Negative
Mirtazapine	Positive
Quetiapine	Negative
Zopiclone	Negative
Clonazepam	Negative
Fentanyl	Positive
Alprazolam	Negative
Etizolam	Negative
Morphine	Positive
Codeine	Positive
Norbuprenorphine	Negative
Benzoylecgonine (Coc Met)	Positive
Nordiazepam	Positive
Dihydrocodeine	Negative
Oxazepam	Negative
Diazepam	Positive
Temazepam	Negative
Methadone metabolite (EDDP)	Positive
Heroin metabolite (6-MAM)	Negative
Lorazepam	Negative
Amphetamine	Negative
Buprenorphine	Negative
MDMA (ecstasy)	Negative
Methadone	Positive

**Figure 3:**
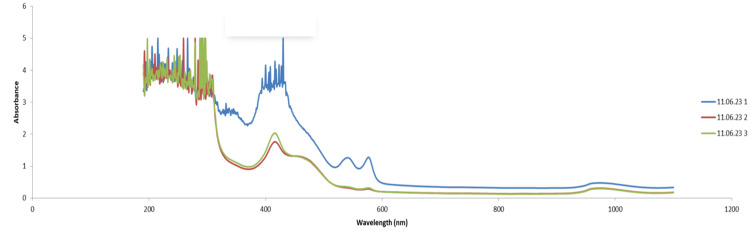
Absorption spectra of blood samples obtained from the patient

Samples taken from the patient were sent to the biochemistry laboratory to scan the absorbance of the three serum samples and compare them to a drug-free serum to look for any peaks and to determine if there is a possibility that any of these drugs of abuse may have caused the interference. In Figure 3 (blue line), it was discovered that the first sample received by the laboratory exhibited a significant peak around 400 nm, which corresponds to the wavelengths utilized for blood gas bilirubin measurement. This occurrence likely contributed to the false-positive result in the blood gas bilirubin.

Interestingly, the subsequent samples collected on the same day (represented by the green and red lines in Figure 3) showed a significantly reduced absorbance in this region. The time gap between the initial and follow-up samples was six hours. This pronounced peak followed by a rapid decline in absorbance could potentially be explained by the presence of a drug with a short half-life that the patient had taken.

**Figure 4:**
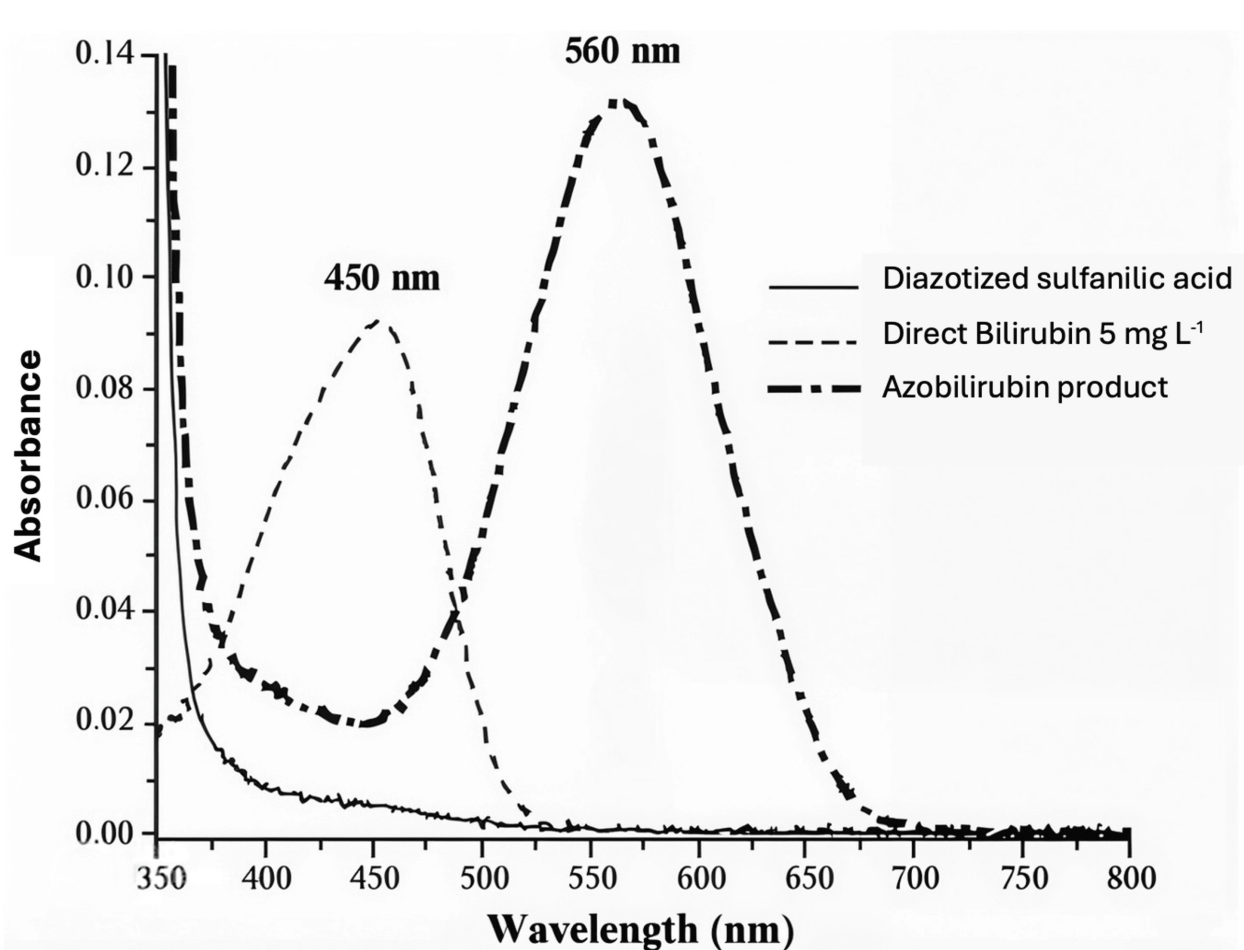
Absorption spectra of bilirubin

**Figure 5:**
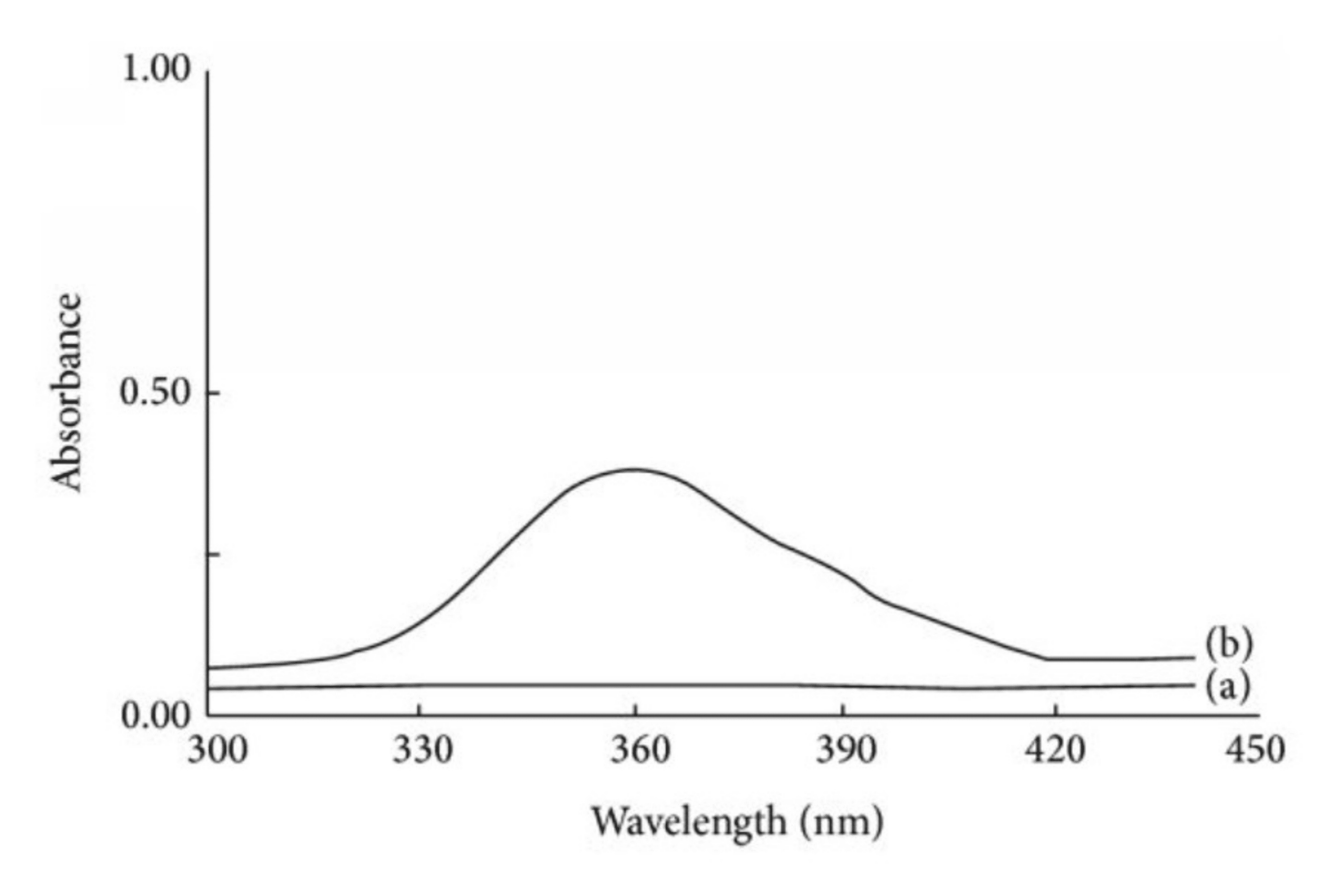
Absorption spectra of pregabalin

**Figure 6:**
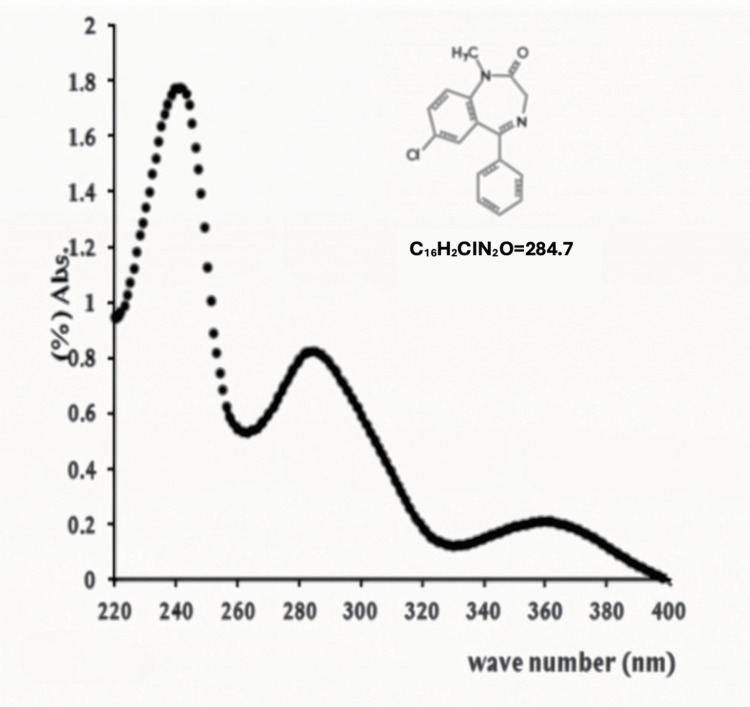
Absorption spectra of diazepam

Oral pregabalin has an elimination half-life of approximately six hours [3], while oral diazepam reaches peak plasma concentrations within 1 to 1.5 hours, followed by a prolonged terminal elimination phase with a half-life of approximately 48 hours [4]. The absorbance spectra of diazepam and pregabalin overlap with bilirubin in the 350-500 nm range, as illustrated in Figure 4, Figure 5, and Figure 6.

Pregabalin is not metabolized into any active compounds, whereas diazepam undergoes metabolism into active compounds that absorb light within the 450-500 nm range. These metabolites are suspected contributors to the interference observed in the bilirubin measurement. Therefore, any substance present in the patient’s blood that absorbs light within the 350-500 nm range could potentially lead to a false-positive result. Given the patient’s suspected overdose of diazepam and pregabalin, these compounds may have been responsible for the observed interference.

The patient initially failed a trial of extubation but was successfully extubated after two days. An echocardiogram was performed to assess the pericardial effusion identified on CT, revealing a reduced ejection fraction of 35%, a dilated left atrium, right atrium, and right ventricle, as well as moderate pulmonary hypertension with an RVSP of 43. However, the left ventricle size was normal, and the pericardial effusion had resolved. Following a cardiology review, he was started on bisoprolol and ramipril. He was subsequently transferred from the ICU to the coronary care unit and referred to the drug and alcohol team for further follow-up.

## Discussion

This case raises several important clinical considerations. Elevated serum bilirubin is a common finding in both primary and hospital care settings [2]. In cases of acute hepatic injury, prothrombin time and, to a lesser extent, total bilirubin serve as key indicators of disease severity [3]. Hyperbilirubinemia can result from disruptions at any stage of bilirubin metabolism, including excessive production, impaired liver uptake, defects in conjugation, or biliary excretion abnormalities [2].

While hyperbilirubinemia is frequently observed, it does not always indicate liver dysfunction. Its presence should be interpreted in the broader context of the patient’s history, symptoms, and biochemical markers. The association between sedative drugs like diazepam and pregabalin and false-positive hyperbilirubinemia is uncommon. Although these medications rarely cause liver injury, their potential to influence bilirubin levels is a noteworthy consideration for clinicians, particularly in acute care settings. This underscores the need for a comprehensive diagnostic approach that integrates laboratory findings with clinical context to prevent misdiagnosis and unnecessary interventions.

Bilirubin can be measured using several methods, including (1) the direct diazo reaction; (2) high-performance liquid chromatography; (3) direct spectrophotometry; and (4) enzymatic methods [4]. According to Wang et al., blood gas analyzers use direct spectrophotometry and support multi-test panels, providing rapid results. As a result, they are commonly used in neonatal ICUs for diagnosing, risk stratifying, and guiding treatment in cases of neonatal hyperbilirubinemia [5].

There are significant differences between blood gas analyzers and laboratory auto-analyzers, particularly regarding the measurement of individual parameters and potential measurement errors. Arterial blood gas point-of-care testing is conducted at or near the patient’s location without requiring sophisticated laboratory equipment [4]. Blood gas analyzers use onboard spectrophotometers to measure absorbance at specific wavelengths, but they are not bilirubin-specific.

Occasionally, abnormal parameter concentrations may arise from errors related to the blood gas analyzer itself (e.g., poor quality control measures) or from interfering substances within the serum. Research by Al Riyami et al. has shown that 0-desmethylnaproxen, a metabolite of naproxen, can also lead to spurious hyperbilirubinemia [6]. Similarly, a study by Wang et al. in neonatal populations - particularly among those at high risk for severe hyperbilirubinemia - demonstrated that bilirubin measurements from Roche blood gas analyzers do not consistently correlate with total serum bilirubin levels [5]. Under standard conditions, blood gas analyzer results may be acceptable, but they are susceptible to non-specific interferences.

Blood gas bilirubin measurement relies on the absorbance of native bilirubin, which has a broad absorbance spectrum ranging from 350 to 500 nm, with a characteristic peak at 450 nm [7]. Due to its strong absorbance in the ultraviolet and visible regions of the electromagnetic spectrum, bilirubin has considerable potential to interfere with spectrophotometric measurements [8]. Additionally, drugs that displace bilirubin from albumin (e.g., sulfonamides) can theoretically alter results by modifying bilirubin’s absorbance spectrum.

In contrast, laboratory-based bilirubin measurement uses the diazo reaction to quantify total bilirubin in serum or plasma. In this method, bilirubin reacts with a diazo reagent in the presence of caffeine, benzoate, and acetate as accelerators to form azobilirubin. The system then measures the change in absorbance, with the diazo-bound bilirubin shifting the peak absorbance to around 560 nm (see Figure 3 for illustration) [3]. This change in absorbance is directly proportional to the bilirubin concentration in the sample [3].

A limitation of this study is that the concentrations of other drugs detected in the urine toxicology screen (e.g., mirtazapine and fentanyl) were not measured. A potential avenue for future research could involve confirming drug-specific interference by measuring the concentrations of these substances to further explore their impact on bilirubin measurement.

## Conclusions

This case highlights the potential for false-positive bilirubin readings on blood gas analysis due to interference from drugs like diazepam and pregabalin. Clinicians should exercise caution and confirm such findings with formal laboratory testing, particularly in patients with a history of substance misuse. Correlating bilirubin levels with clinical signs, such as jaundice, is essential to prevent misdiagnosis. When discrepancies arise, consulting the biochemical laboratory is advisable to ensure accurate diagnosis and appropriate patient management.
